# Malingering assessment after severe traumatic brain injury in forensic psychology with a potential embedded symptom validity indicator of Symptom Checklist 90

**DOI:** 10.3389/fpsyg.2024.1320636

**Published:** 2024-02-08

**Authors:** Cong Liu, Qiuying Lu, Guangxun Rao, Xiaorui Chen, Man Liang, Zilong Liu

**Affiliations:** ^1^Department of Forensic Medicine, Tongji Medical College, Huazhong University of Science and Technology, Wuhan, China; ^2^Department of Forensic Medicine, Shaoxing University Forensic Center, Shaoxing, China

**Keywords:** forensic psychology, traumatic brain injury, malingering, Symptoms Checklist 90, intelligence quotient, P300 event-related potentials, disability compensation

## Abstract

**Objective:**

Malingering of neuropsychological damage is common among traumatic brain injury patients pursuing disability compensation in forensic contexts. There is an urgent need to explore differences in neuropsychological assessment outcomes with different levels of cooperation.

**Methods:**

A total of 420 participants with severe traumatic brain injury were classified into malingering group, partial cooperation group, and complete cooperation group according to the Binomial forced-choice digit memory test. The Wechsler Adult Intelligence Scale, event-related potential component, and Symptom Checklist 90 were applied subsequently to assess the psychological status of participants.

**Results:**

Participants in the malingering group presented lower scores in the binomial forced-choice digit memory test and the Wechsler Adult Intelligence Scale, lower P3 amplitude, and simultaneously higher scores in the Symptom Checklist 90 than the other two groups. The actual intelligence quotient of participants with malingering tendencies ranged mostly between normal and marginal damage, and they often reported elevated whole scale scores in the Symptom Checklist 90. The Cooperation Index (defined as the ratio of positive symptom distress index to global severity index, CI) was proposed and validated to function as an embedded validity indicator of the Symptom Checklist 90, and the area under the receiver operating characteristic (ROC) curve was 0.938. When valued at 1.28, CI has the highest classification ability in differentiating malingering from non-malingering. Combined with the CI and P3 amplitude, the area under the ROC curve for malingering diagnosis further reached 0.952.

**Conclusion:**

Any non-optimal effort in a forensic context will lead to unexpected deviation in psychology evaluation results. CI is a potential candidate to act as an embedded validity indicator of the Symptom Checklist 90. The combination of CI and P3 amplitude can help to identify malingering in participants after severe traumatic brain injury.

## 1 Introduction

Traumatic brain injury (TBI) is one of the most common forms of injury in clinical settings. Its prognosis is heterogeneous due to the different severity and brain sites implicated in the injury, varying from being able to live independently to a vegetative state and even death, bringing huge burdens to individuals, families, and society. In addition to some non-specific symptoms such as headaches, fatigue, dizziness, and sensitivity to light and sound (Bryant, 2011), a number of survivors often developed various neuropsychological sequelae, manifested as cognitive impairment (attention and concentration, verbal and visuospatial memory, naming, orientation and insight, executive function, and signal processing speed) (Miotto et al., 2010), mood disorders (anxiety and depressive disorders and dysthymia) (Osborn et al., 2014; Scholten et al., 2016), behavioral problems (apathy, irritability and aggression, and reduction in motivation and self-esteem) (Miotto et al., 2010; Starkstein and Pahissa, 2014), personality change (a combination of behavioral problems and mood disorders) (Max et al., 2015; Barrash et al., 2018), neurosis (compulsions, panic disorder, social phobia, and agoraphobia) (Bryant et al., 2010; Rydon-Grange and Coetzer, 2019), and even psychotic symptoms (hallucinations and delusions) (Fujii and Fujii, 2012), leading to poor community participation and social acceptance (Theadom et al., 2018).

Forensic psychological assessment after TBI contributes a lot to trauma-related disability compensation. Because of the attractive economic compensation, people undergoing forensic psychological evaluation were often prone to be “dishonest” (Hampson et al., 2014; Kanser et al., 2017), manifested as faking, exaggerating, or fabricating symptoms of psychological and cognitive impairment. Given that most post-traumatic symptoms are subjective, easily coached, and simulated, it is challenging for the examiner to judge the authenticity of the symptoms through behavioral observation and psychometric tests (Kanser et al., 2017). Malingering makes trauma-related mental disorders much more confusing.

A variety of psychological measurements and neurophysiological tests have become indispensable tools for forensic psychological evaluation after TBI. In China, the most common tests for forensic disability assessment include the P300 event-related potentials (ERPs), the Wechsler Intelligence Scale for Adult Chinese Revised (WAIS-RC), and the Symptom Checklist 90 (SCL-90). In some measures, validity scales/tests are embedded to help physicians determine whether the participants’ performance is trustworthy, such as the F scale in the Minnesota Polyphasic Personality Scale-2 (MMPI-2) (Baer et al., 1999) and the Digit Span subtest in WAIS (Webber and Soble, 2018; On et al., 2020). The SCL-90 is routinely used as part of the test batteries for cognitive status and neuropsychological impairment evaluation (Bolzenius et al., 2018; Sigurdardottir et al., 2020) and is a time-sensitive screening measure in forensic settings. The SCL-90 consists of 90 items, mainly covering nine subscales, including somatization (Som), obsessive-compulsive (OC), interpersonal sensitivity (IS), depression (Dep), anxiety (Anx), hostility (Hos), phobic anxiety (Phob), paranoid ideation (PI), and psychoticism (Psy). There are five alternatives in each item divided according to severity, and the more serious the self-reported symptoms were, the higher each item was scored. The positive symptom total (PST) is recommended to assess malingering (Derogatis, 1992; Sullivan and King, 2010), but its application in forensic practice for malingering assessment is limited because the severity of positive symptoms is not considered. Therefore, it is urgent and necessary to develop an embedded validity indicator (EVI) of SCL-90 to identify invalid responses in a forensic context. In addition, there has been more research on malingering after mild TBI (Silver, 2012; Kirkwood, 2015; Elias et al., 2019; Donders et al., 2021), but less attention has been paid to the symptom validity reported by patients with severe TBI. This study aims to clarify the differences in the results of multiple psychological tests, including WAIS, SCL-90, and ERPs, after severe TBI under different cooperation degrees and try to find a way to make the assessments, especially the SCL-90, more objective and reliable, which has important practical significance in the forensic context.

## 2 Materials and methods

### 2.1 Participants

A total of 420 participants (319 men and 101 women) with severe TBI (STBI) who participated in this study were recruited from the Tongji Medicolegal Expertise Center in Hubei Province, China. The study protocol was approved by the Ethics Committee of the University, and the personal information of all the participants was strictly confidential. The following inclusion criteria applied to all the participants (Liu et al., 2016): (1) aged over 16 years old; (2) TBI occurred at least 6–12 months before the present study; (3) there were traumatic imaging findings of brain damage; (4) The Glasgow Coma Scale scores (GCSs) ranged from 3 to 8 in the first 6 h after TBI without the use of sedatives and paralytics; (5) psychological complaints remained after the end of clinical treatment; and (6) involved external incentives. The exclusion criteria included the following: (1) individuals who were unable to communicate with or complete inspection items due to conscious disturbance, severe intellectual impairment, visual and hearing impairments, and aphasia; (2) history of brain injury or psychosis before the current STBI; and (3) history of use of psychoactive substances and antipsychotics.

### 2.2 Instruments

#### 2.2.1 Binomial forced-choice digit memory test

The effort test is a typical memory test relying mainly on the instantaneous memory ability, which is generally not affected by the severity of brain injury (Hampson et al., 2014). The binomial forced-choice digit memory test (BFDMT) is one kind of effort test to assess if the participants are putting forth a good effort in forensic psychological assessment. As a revised version of the Hiscock Digital Memory Test (DMT), it was developed and widely used in China (Liu et al., 2016; Wang et al., 2017; Zhong et al., 2021). Our previous study have shown that BFDMT has a high accuracy in identifying post-traumatic malingering (Liu et al., 2016). The test has 24 items in total, with easy items and difficult items accounting for half. Each item has a five-digit number stimulus card and two five-digit number recognition cards; only one five-digit number in the recognition card is identical to the anterior stimulus card, and another is similar to or distinctly different from the anterior one. The stimulus card was presented for 5 s, and then the participant was required to pick out the five-digit number in the recognition card identical to the anterior stimulus card. This test can be completed on the computer in several minutes. For analysis, the participants were divided into three groups based on their total score on the test: scores below 18 for the malingering group (M-G), scores between 18 and 21 for the partial cooperation group (PC-G), and scores above 21 for complete cooperation group (CC-G). Given that obvious external incentives exist in the forensic context, we used BFDMT to screen participants who have an intentionally dishonest response for an expected return in our research.

#### 2.2.2 Wechsler Adult Intelligence Scale

The Wechsler Intelligence Scale for Adult Chinese Revised (WAIS-RC) was applied in this study to assess the full-scale intelligence quotient (FIQ) of participants. Studies have shown that WAIS was a useful clinical tool for assessing cognitive impairment in participants with mild, moderate, or severe TBI (Carlozzi et al., 2015).

#### 2.2.3 P300 event-related potentials

The classic auditory “Odd-ball” paradigm was used in the present study to record the ERPs, including N2 and P3 components. All participants were presented with a random series of tones via high-fidelity earphones, a high-frequency tone of 2000 Hz (probability: 20%) as the target stimulus, and a low-frequency tone of 1,000 Hz (probability: 80%) as the standard stimulus. Both tones were present at 80 dB for 50 ms each time at an interval of 1,500 ms ± 100 ms, and the task was to press a button only when the target tone was given. For each participant, the acoustic stimulation was repeated 100 times per test, and the test was repeated three times. The electroencephalographic activity was recorded by the United States 37-lead Neuro-scan ERP computer system with silver electrodes placed at Cz for record and Fz for the ground wire; two earlobe electrodes were used as a reference, and all electrode impedances were below 5 KΩ. The operation was carried out in the electromagnetic shielding chamber to reduce external interference. Participants were given a certain amount of training before the test and were given a 5-min rest during the interval between the tests. The participants were required to stay relaxed to minimize electroencephalogram contamination. The N2 and P3 components were recorded, and the amplitude and latency were measured for analysis.

#### 2.2.4 Symptom Checklist 90

This quiz was completed on the computer to obtain the sum scores for each subscale, the global score, the global severity index (GSI), the PST, and the positive symptom distress index (PSDI). As a self-report scale, the accuracy of SCL-90 results is closely related to the test status of the participants. Participants who are faking bad symptoms of impairment seem to choose more serious options indiscriminately and widely in the test due to a lack of relevant knowledge, thus causing the PSDI scores to get closer to the GSI scores. To identify this tendency, we defined the ratio of PSDI to GSI as a Cooperation Index (CI), which took both the number and extent of positive symptoms into consideration to act as EVI for invalid responses due to compensation psychology in a forensic context.

### 2.3 Procedure

**Figure 1:**
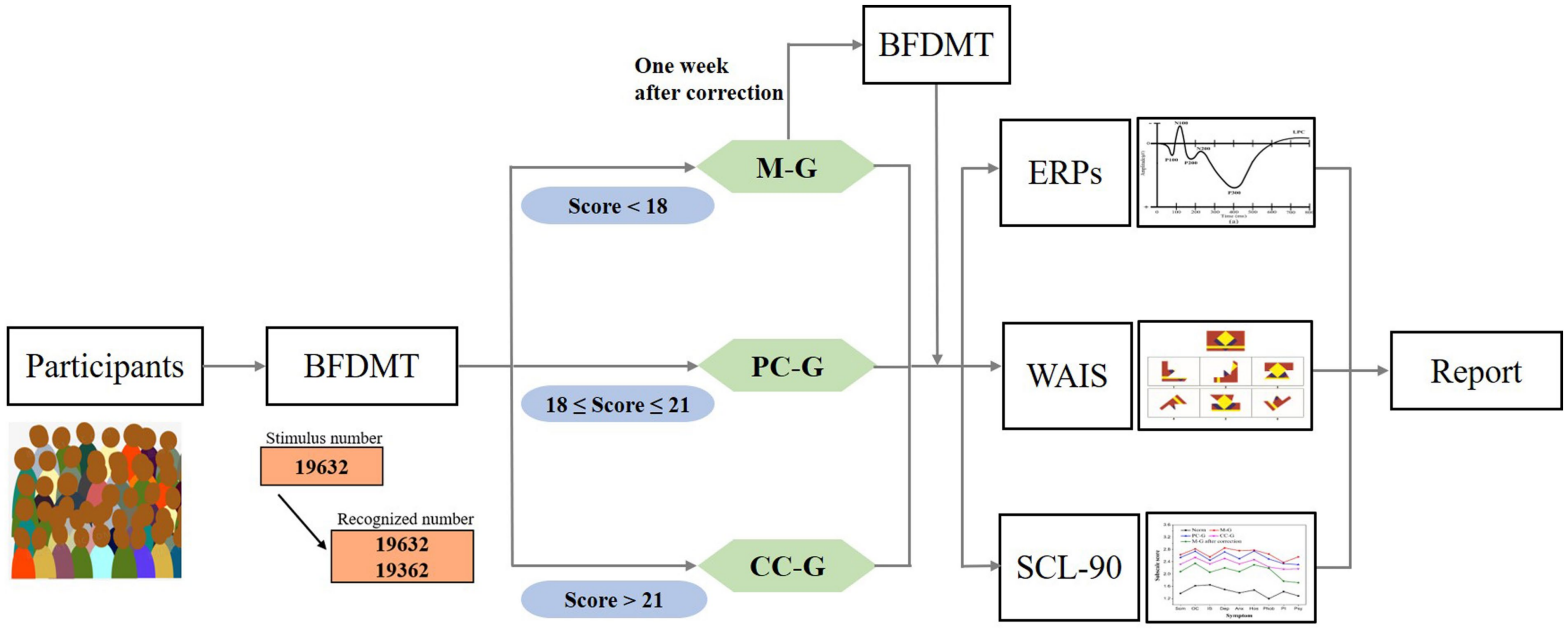
The test procedure for this study.

The procedure of tests for this study is shown in Figure 1. First, the demographic information and medical records were gathered for analysis. Then, the participants were classified into M-G, PC-G, and CC-G according to their BFDMT scores, and all participants were administered the WAIS, SCL-90, and ERPs, respectively. For participants who were in the obviously bad cooperative state (M-G), neurophysiologists tactfully and non-judgmentally presented their inconsistencies throughout the evaluation process and offered a face-saving way out of the interaction to achieve the purpose of correcting malingering. A week after the malingering correction, all the tests were administrated again for re-evaluation.

### 2.4 Statistical analysis

Levene’s test for equality of variances was applied to test the homogeneity of variance in the three groups. A one-way analysis of variance and a non-parametric test were conducted to compare the data between the three groups according to the results of the variance homogeneity test. A paired t-test was conducted to analyze the differences in results of mental impairment before and after malingering correction. *p* < 0.05 was considered statistically significant.

## 3 Results

### 3.1 General demographic data

General demographic data are presented in Table 1. Although the majority of participants were male, no statistical difference regarding sex, age, time post-injury, education years, and GCSs was found among M-G, PC-G, and CC-G (Table 1). This suggested that there was no demographic bias between the groups in this study.

**Table 1:** Comparison of general information between the three groups.

	M-G		PC-G		CC-G	
	M (*n* = 106)	F (*n* = 42)	M (*n* = 105)	F (*n* = 29)	M (*n* = 108)	F (*n* = 30)
Age (year)	43.77 ± 13.13	43.29 ± 12.11	39.90 ± 13.95	39.52 ± 13.98	42.17 ± 14.02	34.33 ± 13.16
Time post injury (month)	9.49 ± 5.28	9.24 ± 4.32	8.33 ± 3.94	9.21 ± 5.49	8.54 ± 5.11	8.10 ± 4.00
Education years	9.02 ± 3.03	9.51 ± 3.60	9.18 ± 2.60	8.85 ± 3.59	9.27 ± 2.65	9.90 ± 3.76
GCSs	6.21 ± 1.32	6.40 ± 1.85	6.38 ± 1.69	6.00 ± 1.23	6.35 ± 1.54	6.40 ± 1.57

M-G, malingering group; PC-G, partial cooperation group; CC-G, complete cooperation group; M, male; F, female. GCSs were evaluated in the first 6 h after traumatic brain injury.

### 3.2 BFDMT data analysis

As expected, the difference in total BFDMT scores between the three groups was statistically significant (*p* < 0.01; Table 2). A non-parametric test showed that the BFDMT scores of M-G were significantly lower than those of PC-G and CC-G. In terms of item types, although the scores on the easy items were very close, difficult items and total scores of PC-G were significantly lower than CC-G (*p* < 0.01). After malingering correction of M-G participants, all the BFDMT scores were significantly increased (*p* < 0.01), the score on easy items reached PC-G and CC-G levels, and the scores on difficult items were close to the other two groups (Table 3).

**Table 2:** Comparison of BFDMT items, FIQ, and ERPs (latency and amplitude of N2 and P3) among M-G, PC-G, and CC-G groups.

	M-G	PC-G	CC-G	*p*
Easy item score	9.05 ± 2.60	11.49 ± 0.77	11.88 ± 0.34	<0.001
Difficult item score	4.82 ± 2.52	8.04 ± 1.17	11.09 ± 0.79	<0.001
Total score	13.66 ± 3.26	19.51 ± 1.56	22.97 ± 0.82	<0.001
FIQ	70.10 ± 8.97	74.31 ± 10.26	80.52 ± 11.39	<0.001
N2 latency	236.59 ± 20.04	239.09 ± 19.97	235.34 ± 18.09	0.349
N2 amplitude	0.18 ± 1.97	0.64 ± 2.04	0.44 ± 2.09	0.301
P3 latency	325.67 ± 16.53	330.51 ± 19.72	324.06 ± 20.51	0.043
P3 amplitude	6.50 ± 2.21	7.18 ± 2.68	7.81 ± 3.04	0.002

M-G, malingering group; PC-G, partial cooperation group; CC-G, complete cooperation group; BFDMT, binomial forced-choice digit memory test; FIQ, Full Scale Intelligence Quotient of Wechsler Adult Intelligence Scale; ERPs, event-related potentials.

**Table 3:** Comparison of BFDMT and SCL-90 items before and after malingering correction.

	Before correction	After correction	*t*	*p*
Easy item score	9.05 ± 2.60	11.70 ± 0.95	3.95	0.003
Difficult item score	4.82 ± 2.52	9.20 ± 1.75	7.12	0.000
Total score of BFDMT	13.66 ± 3.26	20.9 ± 1.85	8.37	0.000
Som	2.63 ± 0.84	2.08 ± 0.79	4.70	0.001
OC	2.82 ± 0.80	2.35 ± 0.48	3.39	0.008
IS	2.56 ± 0.88	2.06 ± 0.64	8.60	0.000
Dep	2.85 ± 0.85	2.20 ± 0.45	5.25	0.001
Anx	2.76 ± 0.93	2.08 ± 0.71	4.59	0.001
Hos	2.78 ± 1.02	2.30 ± 1.01	3.56	0.006
Phob	2.65 ± 1.04	2.19 ± 0.65	4.25	0.002
PI	2.38 ± 0.86	1.77 ± 0.53	4.98	0.001
Psy	2.56 ± 1.30	1.72 ± 0.48	3.06	0.013
global score of SCL-90	240.73 ± 69.40	186.80 ± 46.08	5.27	0.001
GSI	2.70 ± 0.75	2.08 ± 0.51	5.26	0.001
PST	63.17 ± 18.92	57.10 ± 20.59	3.02	0.014
PSDI	3.33 ± 0.64	2.66 ± 0.30	4.31	0.002
CI	1.23 ± 0.28	1.33 ± 0.28	3.02	0.027

BFDMT, binomial forced-choice digit memory test; SCL-90, Symptom Checklist 90; Som., somatization; OC, obsessive-compulsive; IS, interpersonal sensitivity; Dep, depression; Anx, anxiety; Hos, hostility; Phob, phobic anxiety; PI, paranoid ideation; Psy, psychoticism; GSI, global severity index; PST, the positive symptom total; PSDI, the positive symptom distress index; CI, cooperation index.

### 3.3 WAIS data analysis

The measured FIQ was the lowest in M-G (70.10 ± 8.97) and the highest in CC-G (80.52 ± 11.39), and the difference among the three groups was statistically significant (Table 2; *p* < 0.01). After the malingering correction of M-G participants, the FIQ (84.00 ± 10.36) increased by 20% compared to before the correction.

### 3.4 Data analysis of ERPs

**Figure 2:**
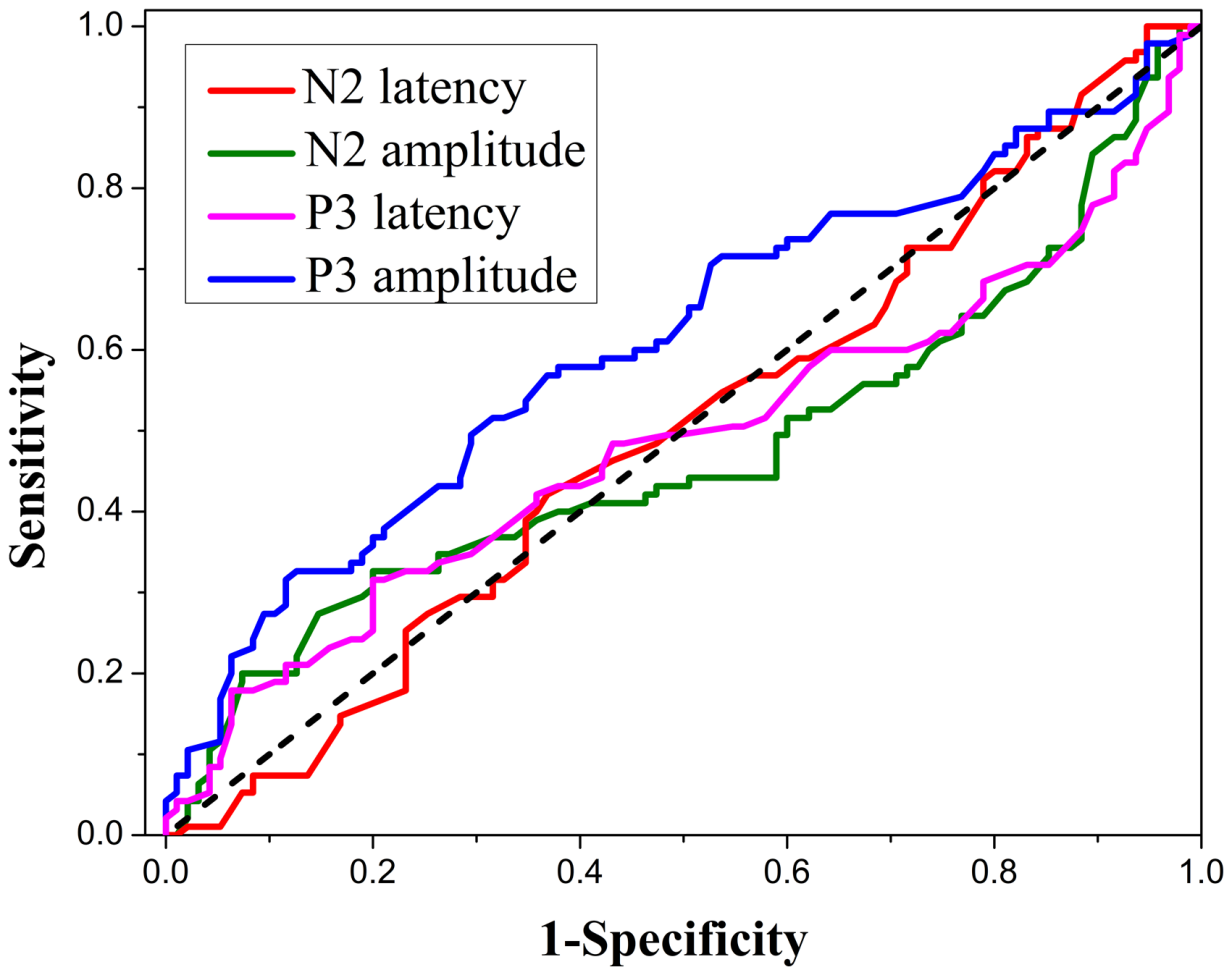
The receiver operating characteristic (ROC) analysis of N2 latency, N2 amplitude, P3 latency, and P3 amplitude as indicators for malingering assessment.

There was no significant difference in N2 latency and amplitude between the three groups (Table 2). However, a significant difference was found in P3 amplitude between M-G and CC-G (*p* < 0.01). The FIQ has a negative correlation with P3 latency, and the correlation coefficients were −0.283, −0.390, and −0.538 in M-G, PC-G, and CC-G, respectively (*p* < 0.01, each). The better the participants performed on the effort test, the lower the average P3 latency, while the correlation between FIQ and P3 latency was higher, likely due to their greater concentration. The FIQ was positively correlated with P3 amplitude, and the correlation coefficients were 0.223, 0.215, and 0.175 in M-G, PC-G, and CC-G, respectively (*p* < 0.01, each). Compared with N2 latency and N2 amplitude, P3 latency and P3 amplitude had a certain effect in distinguishing malingering, and the area under the receiver operating characteristic (ROC) curve was 0.611 (Figure 2).

### 3.5 SCL-90 data analysis

**Figure 3:**
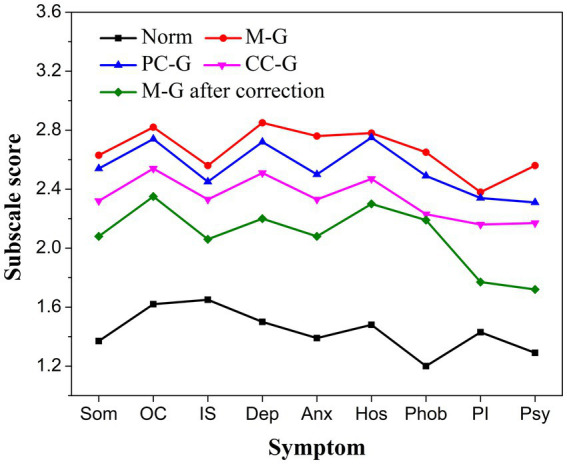
The nine subscale scores of SCL-90 of patients from five groups (norm, M-G, PC-G, CC-G, and M-G after correction).

All the nine subscale scores, the global score, GSI, PST, and PSDI in the three STBI groups were significantly higher than the norm (Jin et al., 1986; Table 4; Figure 3). Among the three groups, the M-G scored the highest, the CC-G scored the lowest, and the difference in most items was extremely significant (*p* < 0.01) except IS, Hos, and PI (*p* < 0.05). Compared to CC-G, the Som, OC, Hos, Phob, GSI, PST, and PSDI in PC-G were higher (*p* < 0.05). The global score in PC-G also showed an uptrend compared to CC-G (*p* = 0.057). After corrective malingering measures were applied, subscale scores in the M-G group declined significantly overall (Table 3; Figure 3), which laterally demonstrated the success of BFDMT classification in malingering or not.

**Table 4:** Comparison of the mean scores of SCL-90 items.

	Norm	M-G	PC-G	CC-G	*p*
Som.	1.37 ± 0.48	2.63 ± 0.84	2.54 ± 0.88	2.32 ± 0.87	0.011
OC	1.62 ± 0.58	2.82 ± 0.80	2.74 ± 0.84	2.54 ± 0.87	0.017
IS	1.65 ± 0.51	2.56 ± 0.88	2.45 ± 0.91	2.33 ± 0.98	0.031
Dep	1.50 ± 0.59	2.85 ± 0.85	2.72 ± 0.97	2.51 ± 0.96	0.010
Anx	1.39 ± 0.43	2.76 ± 0.93	2.50 ± 0.94	2.33 ± 0.98	0.001
Hos	1.48 ± 0.56	2.78 ± 1.02	2.75 ± 1.05	2.47 ± 1.06	0.022
Phob	1.20 ± 0.41	2.65 ± 1.04	2.49 ± 1.03	2.23 ± 1.08	0.003
PI	1.43 ± 0.57	2.38 ± 0.86	2.34 ± 0.99	2.16 ± 0.97	0.048
Psy	1.29 ± 0.42	2.56 ± 1.30	2.31 ± 0.86	2.17 ± 0.87	0.007
Global score	129.96 ± 38.76	240.73 ± 69.40	229.21 ± 72.98	212.25 ± 77.74	0.005
GSI	1.44 ± 0.43	2.70 ± 0.75	2.56 ± 0.83	2.34 ± 0.86	0.002
PST	24.92 ± 18.41	63.17 ± 18.92	59.76 ± 22.97	54.09 ± 23.86	0.002
PSDI	2.60 ± 0.59	3.33 ± 0.64	3.25 ± 0.64	3.08 ± 0.65	0.004
CI	1.81 ± 0.32	1.23 ± 0.28	1.31 ± 0.31	1.41 ± 0.33	0.001

M-G, malingering group; PC-G, partial cooperation group; CC-G, complete cooperation group; SCL-90, Symptom Checklist 90; Som, somatization; OC, obsessive-compulsive; IS, interpersonal sensitivity; Dep, depression; Anx, anxiety; Hos, hostility; Phob, phobic anxiety; PI, paranoid ideation; Psy, psychoticism; GSI, global severity index; PST, the positive symptom total; PSDI, the positive symptom distress index.

**Figure 4:**
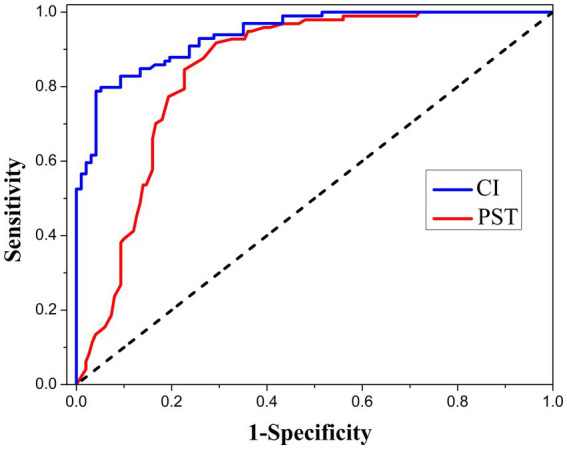
The ROC analysis of cooperation index (CI) and positive symptom total (PST) as validity indicators for malingering assessment in the current sample.

The average CI of concern in this study was 1.81 in the norm, 1.41 in CC-G, 1.31 in PC-G, and 1.23 in M-G. The difference between the three experimental groups was statistically significant (*p* < 0.01). After malingering correction in participants with low BFDMT scores, all the scores of SCL-90 items were significantly decreased (Table 3; *p* < 0.01), and CI increased from 1.23 to 1.33. An ROC analysis was performed to determine the cutoff value with the highest capabilities distinguishing malingering from non-malingering. As shown in Figure 4, the area under the curve of CI reached 0.938 (*p* < 0.01), which was higher than the area under the curve of PST (0.852), indicating that CI is a potential candidate to act as an embedded validity indicator in identifying invalid responses. The diagnostic threshold corresponding to different combinations of sensitivity and specificity is presented in Table 5. When valued at 1.28, CI has the highest classification ability, and the sensitivity and specificity were 0.96 and 0.77, respectively.

**Table 5:** Sensitivity and specificity of malingering diagnosis based on CI.

Cutoff	Sens.	Spec.	Cutoff	Sens.	Spec.
1.07	0.22	1.00	1.60	1.00	0.35
1.10	0.38	1.00	1.70	1.00	0.26
1.20	0.76	0.89	1.80	1.00	0.20
1.21	0.76	0.87	1.90	1.00	0.12
1.22	0.81	0.84	2.00	1.00	0.09
1.23	0.87	0.83	2.10	1.00	0.08
1.24	0.89	0.81	2.20	1.00	0.07
1.25	0.91	0.80	2.30	1.00	0.05
1.26	0.92	0.79	2.40	1.00	0.03
1.28	0.96	0.77	2.60	1.00	0.02
1.29	0.96	0.75	2.80	1.00	0.02
1.30	0.96	0.73	3.00	1.00	0.01
1.40	0.97	0.56	3.20	1.00	0.01
1.50	1.00	0.454	4.40	1.00	0.00

SCL-90, Symptom Checklist 90; Sens., sensitivity; Spec., specificity.

## 4 Discussion

In this study, 420 participants with STBI were divided into three groups according to the BFDMT test results: CC-G, PC-G, and M-G, and their effort status declined successively. The absence of significant differences in the general data regarding sex, age, education, and time post-injury between the three groups offered a good basis for comparison (Table 1). Our findings showed that the WAIS, ERPs, and SCL-90 results were fairly inequable under different effort states. Participants with malingering tendencies usually performed worse in neurocognitive tests, reported more postinjury symptoms with greater severity in structured interviews, and scored higher in self-report scales. Classical neuropsychological assessment has a certain subjectivity; many researchers have devoted themselves to the study of validity tests to detect invalid cognitive performance after TBI (Lippa et al., 2018; On et al., 2020; Kanser et al., 2022). Lippa et al. (2018) found that participants who performed worse on performance validity tests tended to perform worse on cognitive tests and report more symptoms when there was a similar external incentive in a study of 164 veterans with a history of mild TBI. In the present research, all of our efforts aimed at exploring the influence of poor effort state due to external incentives in STBI participants in the forensic context, and we would like to construct an EVI to identify invalid self-report cognitive, somatic, or psychiatric symptoms.

On the BFDMT test, M-G scored an average of 9.05 on the easy items, with little difference from the PC-G and CC-G groups (average 11.49 and 11.88). On the contrary, scores on difficult items vary greatly under different cooperation degrees (ranging from 4.82 ± 2.52 to 11.09 ± 0.79). After malingering correction, the increase of scores on difficult items (average 4.38) gets much higher than that on easy items (average 2.65). All these data (Tables 2, 3) suggested that in the digital forced-type effort test, malingering was more likely to occur in difficult items; in other words, scores of difficult items were more sensitive than those of easy items in recognizing a poor effort.

In most cases, malingering not only exists in participants with poor BFDMT scores below chance level but also in participants who complained of complete memory loss. The forced-choice effort test was closely related to the attention state of the participants and was so simple that even people with substantial cognitive impairment could complete it (Hampson et al., 2014). A statistically correct rate of approximately 50% will be achieved by simply guessing at the alternative items; the correct rate can even reach 78% in TBI patients without financial incentives (Rosen and Powel, 2003). In fact, all the BFDMT scores improved after malingering correction (Table 3).

ERPs, a summation of neurophysiological activities from widely distributed brain areas, were considered to reflect the basic time of central information processing, and accurate records were helpful in monitoring the real-time electrical activity of the brain (Wronka et al., 2013). N2 and P3 are important ERP components associated with cognitive function. N2 represents the perceptual registration following the earlier classification of the stimulus, and P3 is related to attentional resource allocation (Spikman et al., 2004; Wronka et al., 2013). We found no significant difference in N2 components among M-G, PC-G, and CC-G in the present study, but significant differences were found in P3 amplitude and latency (Table 2). This suggested that P3 may be more capable of distinguishing malingering in STBI than N2. Further analysis of the correlation between FIQ and P3 components among the three groups showed that FIQ has an undesirable low positive correlation with P3 amplitude but moderately negatively correlated with P3 latency, and the correlation coefficient was proportional with cooperation degree. The more the participants cooperate, the more the P3 latency can represent the actual FIQ of the participants. In addition, from the results of P3 amplitude in the three groups, it was inferred that the higher the degree of malingering, the lower the P3 amplitude, which was consistent with previous studies (Zhao et al., 2013; Robinson and McFadden, 2020). As a separate indicator of malingering assessment, P3 amplitude has limited efficacy, with an area under the ROC curve of 0.611.

Cognitive impairment, a common neurological sequela of TBI, can lead to poor performance in neuropsychological tests. As an important aspect of cognitive function, FIQ can also be implicated in TBI. A meta-analysis including 81 articles and 3,890 patients in sum revealed that the IQ impairment was proportional to the severity of TBI, and a large number of TBI patients developed FIQ impairment both in the subacute and chronic phase after TBI (Königs et al., 2016). Beyond that, Curtis and colleagues also believe that WAIS indexes have commendable classification accuracy in identifying malingering (Curtis et al., 2009). However, there was a seemingly contradictory phenomenon in our present study in all the participants with STBI; in the absence of malingering, the FIQ ranged widely from nearly normal to marginal damage and mild intellectual disability. Objectively speaking, this contradiction was not incomprehensible. In addition to the poor effort due to interests pursued (Shin et al., 2010; Silver, 2012), the bad influences on different aspects of cognitive functions, including IQ (executive functions, information processing speed, attention and visuomotor functioning, memory, naming, and verbal knowledge), are related to the diverse localization of brain lesions (Miotto et al., 2010; Königs et al., 2016). As the cases were collected, the injured areas of the brain varied among the included participants.

The FIQ was the lowest in M-G (mean 70.10 ± 8.97) and the highest in CC-G (mean 80.52 ± 11.39). For suspected malingerers, the FIQ even reached the value (mean 83.88 ± 10.59) higher than CC-G after correction. Furthermore, considering that M-G had the shortest P3 latency, we have reason to believe that the actual FIQ of the participants in M-G was probably in marginal damage, even with normal status. The probable reason for this phenomenon may be that the participants with relatively mild consequences of intelligence damage were in a nearly complete state of self-awareness and orientation. They can clearly understand what the ongoing tests mean for themselves and pretend to perform poorly to disguise themselves as suffering in a bad state to get more economic compensation in the proceedings. On the contrary, participants with more serious consequences of intelligence damage often be unaware to disguise or easily be disclosed.

A similar phenomenon appeared in the SCL-90 test, which is a worldwide scale used in screening non-patients and conscripts with malingering tendencies (Vetter et al., 2009). Brain trauma does cause neuropsychological symptoms; however, when it comes to personal benefits, scores at self-reporting scales tend to be higher in mild brain injury than the more severe ones (Silver, 2012; Hampson et al., 2014). Similar to previous studies (Vetter et al., 2009; Sullivan and King, 2010), all the SCL-90 scores in the present study were higher than the norm (Jin et al., 1986), and the M-G scored the highest, while the CC-G scored the lowest.

Emotional problems were common and prolonged sequelae following TBI. The long-term prevalence of anxiety disorder and depressive disorder, which often emerged at the same time with each other after TBI, was 36 and 43%, respectively (Scholten et al., 2016). Compared to PC-G and CC-G, the significantly higher Anx and Psy in M-G suggested that STBI participants with a relatively high FIQ in the M-G experienced more anxiety. In addition, the PC-G showed more severe subjective symptoms on Som, OC, Host, and Phob than the CC-G. This means that participants with malingering tendencies prefer to fake bad symptoms about these aspects, which may be unconscious (or passive) exaggeration due to a defense mechanism (for example, the conversation of psychological confliction into somatic symptoms, that is, Som) or simply because of how easily the symptom can be simulated (Shin et al., 2010; Sullivan and King, 2010). These results were consistent with the previous findings that psychopathologies such as posttraumatic stress disorder, somatic complaints, depression, psychoticism, and phobic anxiety were more susceptible to malingering (Sullivan and King, 2010). The elevated self-reported OC was associated with cognitive flexibility and visual memory impairment, and it may be a strategy to respond to poor memory following TBI (Rydon-Grange and Coetzer, 2019).

We found in practice that participants with more serious brain damage consequences confirmed by imaging in PC-G and CC-G usually reported no abnormalities, although they were not in optimistic status, and they rarely complained. Conversely, there was a profile of a wide range of abnormalities from moderate to serious in the mild-damage ones in most subscales. This may not be surprising, since in addition to psychological factors, other factors (e.g., recovery expectation, stereotype threat, negative injury perceptions, stress of pain, hospital procedures, litigation involvement, and disability evaluation) can also play a role in neuropsychological testing (Shin et al., 2010; Silver, 2012; Osborn et al., 2014; Wortzel and Granacher, 2015). On one hand, compared with mild TBI, participants with more severe TBI were confident that they would be compensated, and the reduction in motivation to exaggerate can reduce the number of positive symptoms endorsed on the self-report scales (Starkstein and Pahissa, 2014). On the other hand, in more serious TBI cases, cognitive impairment can lead to a poor self-awareness of symptoms by affecting the encoding ability of traumatic experiences (Bryant, 2011).

SCL-90 has become a favored assessment tool because of its simplicity, comprehensiveness, and relatively low time cost in forensic applications. However, when exposed to situations with obvious external incentives, examiners usually have trouble stopping the pretenders from exaggerating or fabricating symptoms. The test manual recommended a PST score of >50 for men and >60 for women as thresholds for assessing malingering. However, PST only reflected the number of positive symptoms but did not consider the degree of positive symptoms, which limited the practical value (Sullivan and King, 2010). It is meaningful and urgent to propose a new EVI to make SCL-90 more suitable for the forensic context. Taking both the number and degree of positive symptoms into consideration, we defined CI as the ratio of PSDI and GSI to indicate whether the participant was honest in the assessment. The CI of the general population was as high as 1.81, while the CI of cooperative STBI participants (CC-G) was 1.41, and the CI of part cooperative participants (PC-G) was reduced to 1.31 and even more pronounced to 1.23 in STBI participants with malingering tendencies (M-G). When participants picked the most severe of all symptoms without thinking, the CI value was 1. CI showed excellent diagnostic efficiency against malingering with an area under the ROC curve of 0.938, higher than that of PST (0.852). When the threshold was set to 1.28, CI had the highest efficacy in identifying malingering. In this study, for malingerers, CI increased from 1.23 to 1.33 after malingering correction and retesting. All these data suggested that CI is a promising EVI for SCL-90.

**Figure 5:**
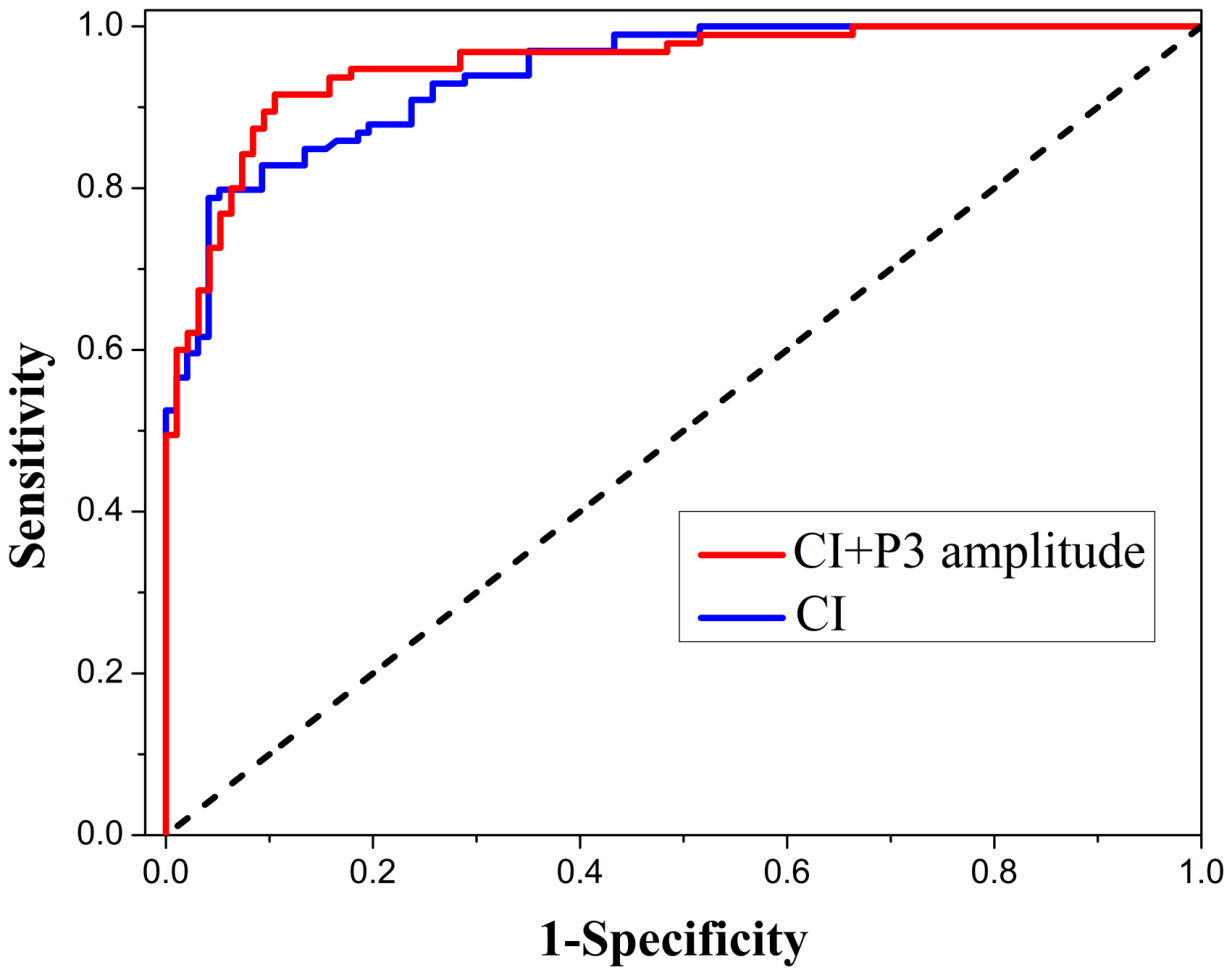
ROC analysis using the combination of CI and P3 latency as the index of malingering assessment in the current sample.

SCL-90 is a self-rating scale, and ERP is an electrophysiologic test. We further used the combination of CI and P3 amplitude to diagnose malingering and found that the classification ability was further improved. The ROC curve is shown in Figure 5. The area under the curve was 0.952, and the sensitivity and specificity reached 0.916 and 0.895 when the Yoden index was maximum.

In conclusion, this study discussed the characteristics and differences in the performance of WAIS, ERPs, and SCL-90 in patients with severe TBI at different levels of effort and proposed for the first time that CI could be used as a potential EVI for SCL-90. The combination of CI and P3 amplitude has outstanding efficacy in diagnosing malingering. To be honest, the exploration of CI in this study has several limitations: (1) All of the participants in our study were STBI participants exposed to explicit external incentives, and several control groups (e.g., STBI patients without external incentives, other traumatic orthopedic injury patients, and simulators) will be set up in our further research to explore the performance of CI in different scenarios; (2) The applicability of CI in mild and medium TBI and its correlation with brain areas of TBI still need further study. In addition, it should be noted that the conclusion of this research is currently only applicable to patients after STBI with obvious external incentives, and its applicability to patients with endogenous psychosis needs further study.

## Data availability statement

The datasets presented in this article are not readily available because data on patients involved in forensic psychiatric evaluation should be kept confidential. Requests to access the datasets should be directed to ZL, E-mail: liuzilongfy@hust.edu.cn.

## Ethics statement

The studies involving humans were approved by Medical Ethics Committee, Tongji Medical College, Huazhong University of Science and Technology. The studies were conducted in accordance with the local legislation and institutional requirements. The participants provided their written informed consent to participate in this study. Written informed consent was obtained from the individual(s) for the publication of any potentially identifiable images or data included in this article.

## Author contributions

CL: Conceptualization, Funding acquisition, Visualization, Writing – original draft, Writing – review & editing. QL: Data curation, Formal analysis, Investigation, Methodology, Validation, Writing – original draft. GR: Conceptualization, Data curation, Formal analysis, Validation, Writing – review & editing. XC: Conceptualization, Supervision, Writing – review & editing. ML: Conceptualization, Methodology, Resources, Writing – review & editing. ZL: Conceptualization, Funding acquisition, Project administration, Resources, Supervision, Writing – review & editing.
